# Development and validation of a risk factor-based system to predict short-term survival in adult hospitalized patients with COVID-19: a multicenter, retrospective, cohort study

**DOI:** 10.1186/s13054-020-03123-x

**Published:** 2020-07-16

**Authors:** Shuai Zhang, Mengfei Guo, Limin Duan, Feng Wu, Guorong Hu, Zhihui Wang, Qi Huang, Tingting Liao, Juanjuan Xu, Yanling Ma, Zhilei Lv, Wenjing Xiao, Zilin Zhao, Xueyun Tan, Daquan Meng, Shujing Zhang, E Zhou, Zhengrong Yin, Wei Geng, Xuan Wang, Jianchu Zhang, Jianguo Chen, Yu Zhang, Yang Jin

**Affiliations:** 1 Department of Respiratory and Critical Care Medicine, NHC Key Laboratory of Pulmonary Diseases, Union Hospital, Tongji Medical College, Huazhong University of Science and Technology, 1277 Jiefang Avenue, Wuhan, 430022 Hubei China; 2 Department of Respiratory and Critical Care Medicine, The Central Hospital of Wuhan, Tongji Medical College, Huazhong University of Science and Technology, 88 Huangxiaohe Rd., Wuhan, 430022 Hubei China; 3 Department of Scientific Research, Union Hospital, Tongji Medical College, Huazhong University of Science and Technology, 1277 Jiefang Avenue, Wuhan, 430022 Hubei China; 4 Department of Pharmacology, School of Basic Medicine, Tongji Medical College, Huazhong University of Science and Technology, 13# Hangkong Road, Wuhan, 430030 Hubei China; 5 Department of Pharmacy, Union Hospital, Tongji Medical College, Huazhong University of Science and Technology, Wuhan, 430022 Hubei China; 6 Hubei Province Clinical Research Center for Precision Medicine for Critical Illness, Wuhan, 430022 Hubei China

**Keywords:** COVID-19, Risk factor, Mortality, Prediction system

## Abstract

**Background:**

Coronavirus disease 2019 (COVID-19) has become a public health emergency of global concern. We aimed to explore the risk factors of 14-day and 28-day mortality and develop a model for predicting 14-day and 28-day survival probability among adult hospitalized patients with COVID-19.

**Methods:**

In this multicenter, retrospective, cohort study, we examined 828 hospitalized patients with confirmed COVID-19 hospitalized in Wuhan Union Hospital and Central Hospital of Wuhan between January 12 and February 9, 2020. Among the 828 patients, 516 and 186 consecutive patients admitted in Wuhan Union Hospital were enrolled in the training cohort and the validation cohort, respectively. A total of 126 patients hospitalized in Central Hospital of Wuhan were enrolled in a second external validation cohort. Demographic, clinical, radiographic, and laboratory measures; treatment; proximate causes of death; and 14-day and 28-day mortality are described. Patients’ data were collected by reviewing the medical records, and their 14-day and 28-day outcomes were followed up.

**Results:**

Of the 828 patients, 146 deaths were recorded until May 18, 2020. In the training set, multivariate Cox regression indicated that older age, lactate dehydrogenase level over 360 U/L, neutrophil-to-lymphocyte ratio higher than 8.0, and direct bilirubin higher than 5.0 μmol/L were independent predictors of 28-day mortality. Nomogram scoring systems for predicting the 14-day and 28-day survival probability of patients with COVID-19 were developed and exhibited strong discrimination and calibration power in the two external validation cohorts (C-index, 0.878 and 0.839).

**Conclusion:**

Older age, high lactate dehydrogenase level, evaluated neutrophil-to-lymphocyte ratio, and high direct bilirubin level were independent predictors of 28-day mortality in adult hospitalized patients with confirmed COVID-19. The nomogram system based on the four factors revealed good discrimination and calibration, suggesting good clinical utility.

## Background

Since December 2019, an ongoing outbreak of coronavirus disease 2019 (COVID-19) has struck Wuhan, Hubei province, China [1–4]. Human-to-human transmission has occurred through respiratory droplets or likely feces [5, 6]. Epidemiological and clinical characteristics of patients with COVID-19 in China have been reported [2–4, 7]. The number of cases grew quickly since January 2020. As of June 9, 2020, 7,039,918 confirmed cases of COVID-19 have occurred, resulting in 404,396 deaths [8].

Outbreaks of COVID-19 infection imposed a great burden on the healthcare system of many countries. To guide the allocation of limited healthcare resources, as well as the timely recognition and intervention of patients who were at high risk of mortality, efficient prognosis of the disease is needed. Previous reports have shown age, Sequential Organ Failure Assessment (SOFA) score, d-dimer, preexisting concurrent cardiovascular or cerebrovascular diseases, amounts of CD3^+^CD8^+^ T cells, and cardiac troponin I to be risk factors for mortality of adult inpatients with COVID-19 [9–11]. Meanwhile, several prognostic models for predicting mortality risk have been developed [12, 13]. The most common predictors included in this prognostic model were age, sex, C-reactive protein (CRP), lactate dehydrogenase (LDH), and lymphocyte count. However, most of these studies have relatively few outcome events, showed a high risk of model overfitting, and failed to clearly describe the intended use of these models.

In this study, we investigated 828 patients with confirmed COVID-19 who were admitted to Wuhan Union Hospital West Area and Central Hospital of Wuhan between January 12 and February 9, 2020. Since the median time to death from illness onset was reported to be 18.5 days, we believed 28-day could be an appropriate time point for the inclusion of mortality events and administrative censoring [10]. We aimed to explore the risk factors of 28-day mortality and develop a nomogram scoring system for predicting 28-day survival probability among patients with COVID-19.

## Methods

### Study design and participants

This multicenter, retrospective, cohort study (clinical trial identifier ChiCTR2000029770) was conducted at Wuhan Union Hospital West Area and Central Hospital of Wuhan. The study was approved by the Institutional Ethics Committee of Union Hospital, Tongji Medical College, Huazhong University of Science and Technology (20200036); the requirement for informed consent was exempted by the Ethics Committee.

**Fig. 1:**
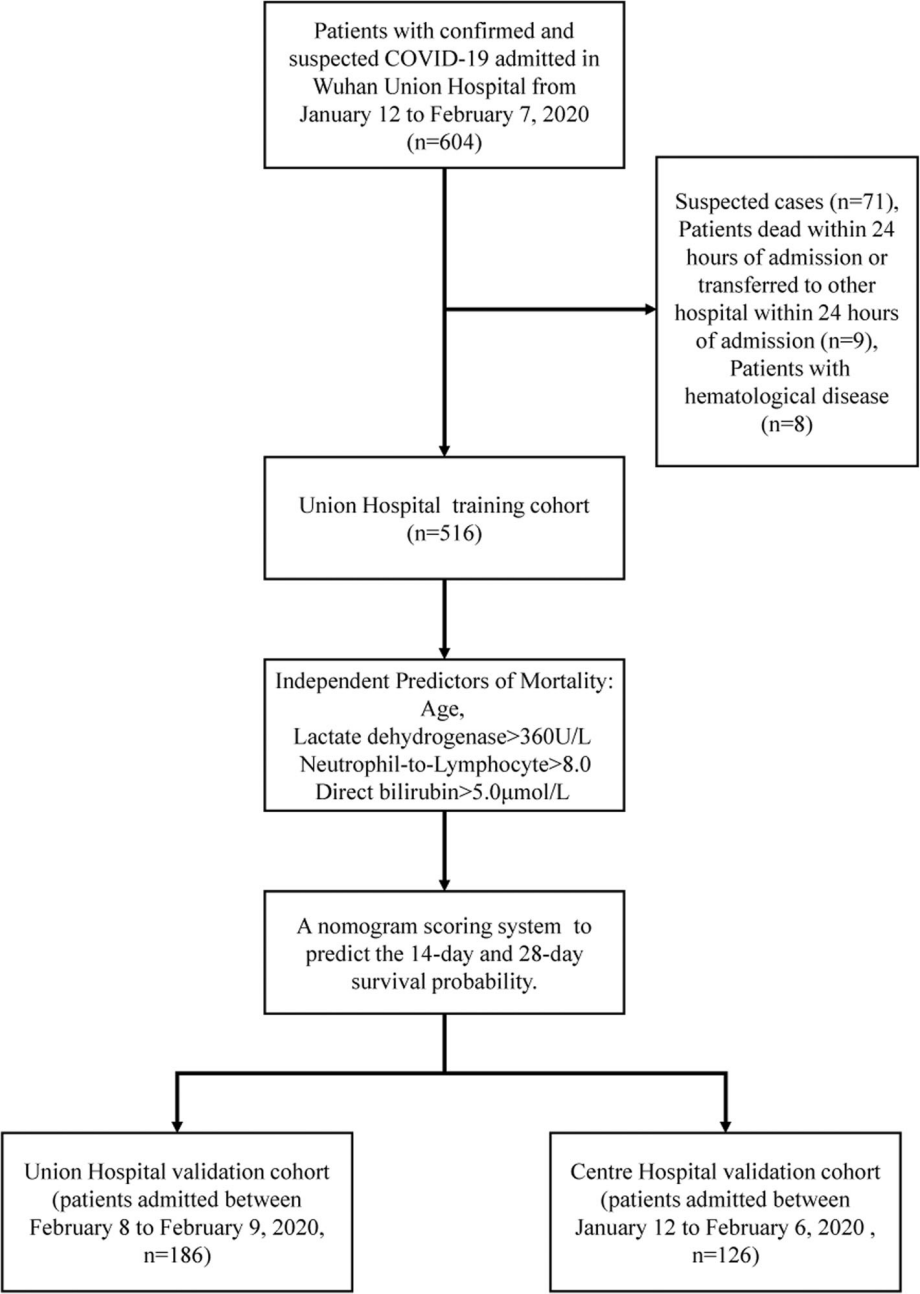
Study flow

The inclusion criterion was adult patients with confirmed COVID-19. Those who lacked laboratory findings and CT images or lost 28-day follow-up were excluded. Besides, patients with hematological diseases had abnormal blood routine test due to their hematologic disorders, which made the analysis of blood routine test unfeasible, and were also excluded. In the training cohort, we retrospectively analyzed 604 consecutive patients with confirmed or suspected COVID-19 who were admitted in Wuhan Union Hospital West Area between January 12, 2020, and February 7, 2020. Eighty-eight of the 604 cases were excluded from the study; among them, 71 were suspected cases, 9 lacked laboratory findings and CT images due to their death or being transferred to other hospitals within 24 h after admission, and 8 patients were with hematological diseases. Finally, a total of 516 patients were enrolled in the training cohort (Union Hospital training cohort, 420 survivors and 96 non-survivors, 87 patients died within 28 days of admission, Fig. 1). Next, another 194 consecutive patients were admitted in Wuhan Union Hospital West Area between February 8, 2020, and February 9, 2020. Among them, 3 were suspected cases, 4 lacked laboratory findings and CT images due to their death within 24 h after admission, and one patient had hematological diseases; 8 patients were excluded from the study. Finally, 186 patients with confirmed COVID-19 were included as external validation cohort 1 (Union Hospital external validation cohort, 156 survivors and 30 non-survivors, 26 patients died within 28 days of admission). A total of 158 patients with confirmed or suspected COVID-19 who were admitted in Central Hospital of Wuhan between January 12, 2020, and February 6, 2020, were selected by simple random sampling. Of the158 patients, 31 were suspected cases and one died within 24 h after admission, all of whom were excluded from the study, and the remaining 126 confirmed patients were included as the external validation cohort 2 (Central Hospital external validation cohort, 106 survivors and 20 non-survivors).

A total of 46 deceased patients had been reported in a previous submission, and 18 patients participated in a phase 3 randomized, double-blind, placebo-controlled, multicenter study for evaluating the efficacy and safety of remdesivir in hospitalized adult patients with severe COVID-19 [14, 15].

### Diagnosis and clinical classification of COVID-19

The diagnosis and clinical classification of COVID-19 were based on the guidelines of the diagnosis and treatment of new coronavirus pneumonia (version 7) published by the National Health Commission of China [16]. The diagnosis was established on the basis of (1) epidemiological history, (2) fever and/or other respiratory symptoms, (3) presence or absence of the imaging findings of novel coronavirus pneumonia, and (4) real-time fluorescence RT-PCR for SARS-CoV-2 nucleic acid which yielded positive results. As for clinical classification, patients were deemed severely ill if they met at least one of the following criteria: (1) shortness of breath, with respiratory rate ≥ 30 breaths/min; (2) oxygen saturation (at resting state) ≤ 93%; (3) PaO_2_/FiO_2_ ≤ 300 mmHg; (4) radiographical imaging showing lesion progression more than 50% within 24–48 h; or (5) respiratory failure, shock, or other organ failures.

### Data collection

Clinicians from the hospital identified patients who satisfied the study inclusion criteria through surveillance of all patients. We collected all available information from patients, their families, physicians, and the electronic medical records in the hospital, including the epidemiological history; clinical, laboratory, and CT findings; treatment (i.e., antiviral therapy, corticosteroid therapy, respiratory support, kidney replacement); and outcomes. All clinical data used in this study were collected from the first day of hospital admission unless indicated otherwise. Electronic medical data were inputted onto a local server. A team of trained physicians searched the patient charts for all the information recorded. For patients discharged within 28 days after admission, patients or their families were followed up to obtain the information about their 14-day and 28-day outcomes by telephone interviews.

### Outcomes

The primary outcome of this study was mortality at 14 days and 28 days after admission.

### Potential prognostic factors

To avoid overfitting in our model, we calculated the numbers of variables allowed to enroll in our multivariable Cox regression model based on a previous study for guidance on sample size requirements for prediction models [17]. In our multivariable model, by setting Nagelkerke’s *R*^2^ = 0.18, we found that our sample size was sufficient to estimate the overall outcome risk and 6 variables could be enrolled in the multivariable analyses. Considering a total number of 516 patients (with 96 decreased patients within 28 days after admission), the final Nagelkerke’s *R*^2^ = 0.163, the Cox-Snell *R* squared statistic (*R*^2^_cs_) = 0.099, and the candidate predictor parameter (EPP) = 14.46, with 95% CI for overall risk = 0.138 and 0.203.

Among a dozen of indicators, which were associated with 28-day mortality in unavailable Cox regression analyses (*P* < 0.001), variables included into the multivariable Cox regression model were selected mainly based on the previous evidence, clinical significance, the correlation between predictors, and availability of data [18]. Previous studies have shown older age, dyspnea, and higher levels of LDH, CRP, and direct bilirubin (DBIL) to be associated with severe disease at admission [19, 20]. Elevated neutrophil-to-lymphocyte ratio (NLR) value was observed in patients who died of COVID-19 and found to be able to predict severe cases of COVID-19 at its early stage [20, 21]. Meanwhile, these risk factors, including older age and higher LDH levels, have been reported to be associated with adverse clinical outcomes in adults with SARS [22, 23]. Other important indicators such as CT images, d-dimer, and ferritin might be unavailable in emergency circumstances. Therefore, we chose age, NLR, LDH, CRP, and DBIL as the five variables for our multivariable Cox regression model. All these variables included in the Cox regression analyses were measured at admission. We converted these indicators including respiratory rate, breaths per minute, NLR, platelets count, alanine aminotransferase (ALT), prothrombin time (PT), and LDH to binary variables and converted these indicators including total bilirubin, white blood cell count, DBIL, urea nitrogen, d-dimer, and CRP to trichotomous variables when performing univariable Cox regression analyses in the training cohort. In addition, variables including LDH and NLR were dichotomized, and direct bilirubin was trichotomous when performing multivariate Cox regression analyses to obtain risk factors for 28-day mortality in the training cohort. These predictors were eventually selected by forward stepwise regression.

### Statistical analysis

Categorical variables were presented as frequency rates and percentages, and continuous variables were expressed as mean ± standard deviation (SD) if they were normally distributed or median (interquartile range [IQR]) if they were not. Proportions for categorical variables were compared using the *χ*^2^ test or Fisher’s exact test. Means for continuous variables were compared using independent group *t* test when the data were normally distributed. Otherwise, the Wilcoxon rank-sum test was employed. 95% confidence interval (CI) of mortality was analyzed by Wilson Score CI.

For the training cohort and the Union Hospital validation cohort, missing data have been mentioned in the relevant tables, and there was no other missing data, unless otherwise noted. And for the Central Hospital external validation cohort, 6 out of 126 missed LDH information, and these missing data were handled by multiple imputations [24].

The nomogram was used to visually score the patients’ various parameters according to the results of multivariable Cox regression analyses, and then to compute the probability of the event based on the patients’ total score. C-index was calculated to evaluate the distinguishing power, and the calibration curve was used to evaluate the calibration of the nomogram. All statistics were two-tailed, and a *P* value less than 0.05 was considered as significant. All statistical analyses were performed by using the SAS software package (version 9.4).

## Results

### Presenting characteristics

The demographic and clinical characteristics at admission for the Union Hospital training cohort (*n* = 516), Union Hospital external validation cohort (*n* = 186), and Central Hospital of Wuhan external validation cohort (*n* = 126) are listed in Table 1. Among the 828 patients, 381 were females and 447 were males. On admission, 289 were mild and 539 were severely ill cases. The median age of non-survivors was older than that of survivors in both 3 cohorts. The median duration from illness onset to admission for all the patients was estimated to be 10 days (IQR, 7.0–13.0), and no difference was seen between the non-survivor and survivor groups (*P* = 0.484). The most common presenting symptoms were fever (704 [85.02%]), cough (565 [68.24%]), and weakness (436 [52.66%]). Other common symptoms included shortness of breath, myalgia, anorexia, dyspnea, diarrhea, and anorexia. A total of 374 (45.17%) patients had chronic diseases; the most common comorbidity was hypertension (259 [31.28%]), followed by diabetes (134 [16.18%]) and chronic cardiac disease (106 [12.80%]). Non-survivors showed more presence of chronic disease than survivors (82/146 vs 292/682, *P* = 0.003).

**Table 1:** Demographic and baseline characteristics of 828 patients with COVID-19

Characteristics		Enrolled patients (*n* = 828)	Training cohort (*n* = 516)		External validation cohort 1 (*n* = 186)		External validation cohort 2 (*n* = 126)	
			Survivors (*n* = 420)	Non-survivors (*n* = 96)	Survivors (*n* = 156)	Non-survivors (*n* = 30)	Survivors (*n* = 106)	Non-survivors (*n* = 20)
Female	*n* (%)	381 (46.01%)	207 (49.29%)	24 (25.00%)	82 (52.56%)	12 (40.00%)	50 (47.17%)	6 (30.00%)
Male	*n* (%)	447 (53.99%)	213 (50.71%)	72 (75.00%)	74 (47.44%)	18 (60.00%)	56 (52.83%)	14 (70.00%)
Age (years)	Median (IQR)	62.0 (51.0–69.0)	59.0 (48.0–67.0)	67.0 (61.0–73.8)	63.0 (52.0–69.0)	68.0 (63.8–76.5)	57 (40.8–67.0)	65.0 (61.5–84.5)
≤ 50	*n* (%)	200 (24.15%)	123 (29.29%)	4 (4.17%)	33 (21.15%)	1 (3.33%)	36 (33.96%)	3 (15.00%)
51–60	*n* (%)	185 (22.34%)	102 (24.29%)	18 (18.75%)	38 (24.36%)	1 (3.33%)	25 (23.58%)	1 (5.00%)
61–70	*n* (%)	276 (33.33%)	118 (28.10%)	41 (42.71%)	57 (36.54%)	16 (53.33%)	36 (33.96%)	8 (40.00%)
> 70	*n* (%)	167 (20.17%)	77 (18.33%)	33 (34.38%)	28 (17.95%)	12 (40.00%)	9 (8.49%)	8 (40.00%)
Symptoms onset to admission, days	Median (IQR)	10.0 (7.0–13.0)	10.0 (7.0–13.0)	10.0 (7.0–13.0)	12.0 (9.0–15.0)	12.0 (7.8–15.0)	7.0 (5.0–10.0)	7.0 (5.3–10.8)
**Symptoms**								
Fever	*n* (%)	704 (85.02%)	374 (89.05%)	80 (83.33%)	128 (82.05%)	20 (66.67%)	85 (80.19%)	17 (85.00%)
Cough	*n* (%)	565 (68.24%)	291 (69.29%)	62 (64.58%)	98 (62.82%)	21 (70.00%)	78 (73.58%)	15 (75.00%)
Weakness	*n* (%)	436 (52.66%)	238 (56.67%)	65 (67.71%)	62 (39.74%)	16 (53.33%)	47 (44.34%)	8 (40.00%)
Shortness of breath	*n* (%)	268 (32.37%)	146 (34.76%)	33 (34.38%)	38 (24.36%)	4 (13.33%)	35 (33.02%)	12 (60.00%)
Dyspnea	*n* (%)	246 (29.71%)	115 (27.38%)	40 (41.67%)	42 (26.92%)	15 (50.00%)	21 (19.81%)	13 (65.00%)
Myalgia	*n* (%)	193 (23.31%)	104 (24.76%)	26 (27.08%)	33 (21.15%)	11 (36.67%)	19 (17.92%)	0
Anorexia	*n* (%)	163 (19.69%)	89 (21.19%)	28 (29.17%)	19 (12.18%)	3 (10.00%)	21 (19.81%)	3 (15.00%)
Diarrhea	*n* (%)	126 (15.22%)	69 (16.43%)	17 (17.71%)	21 (13.46%)	4 (13.33%)	13 (12.26%)	2 (10.00%)
Mild	*n* (%)	289 (34.90%)	134 (31.90%)	0	75 (48.08%)	1 (3.33%)	77 (72.64%)	2 (10.00%)
Severely ill	*n* (%)	539 (65.10%)	286 (68.10%)	96 (100.00%)	81 (51.92%)	29 (96.67%)	29 (27.36%)	18 (90.00%)
**Comorbidities**								
Any	*n* (%)	374 (45.17%)	162 (38.57%)	48 (50.00%)	82 (52.56%)	21 (70.00%)	48 (45.28%)	13 (65.00%)
Hypertension	*n* (%)	259 (31.28%)	107 (25.48%)	31 (32.29%)	53 (33.97%)	16 (53.33%)	35 (33.02%)	12 (60.00%)
Diabetes	*n* (%)	134 (16.18%)	60 (14.29%)	16 (16.67%)	32 (20.51%)	9 (30.00%)	12 (11.32%)	5 (25.00%)
Chronic cardiac disease	*n* (%)	106 (12.80%)	53 (12.62%)	14 (14.58%)	19 (12.18%)	8 (26.67%)	8 (7.55%)	4 (20.00%)
Cerebrovascular disease	*n* (%)	26 (3.14%)	6 (1.43%)	4 (4.17%)	5 (3.21%)	5 (16.67%)	2 (1.89%)	4 (20.00%)
Chronic hepatic disease	*n* (%)	23 (2.78%)	13 (3.10%)	3 (3.13%)	4 (2.56%)	1 (3.33%)	2 (1.89%)	0
Chronic respiratory disease	*n* (%)	25 (3.02%)	11 (2.62%)	3 (3.13%)	8 (5.13%)	0	3 (2.83%)	0
Chronic renal disease	*n* (%)	18 (2.17%)	6 (1.43%)	3 (3.13%)	3 (1.92%)	4 (13.33%)	1 (0.94%)	1 (5.00%)
A history of malignancy	*n* (%)	27 (3.26%)	16 (3.81%)	5 (5.21%)	1 (0.64%)	3 (10.00%)	2 (1.89%)	0

Categorical variables were presented as frequency rates and percentages

Continuous variables were expressed median (IQR)

*CI* confidence interval

### Treatments, outcomes, and complications

**Fig. 2:**
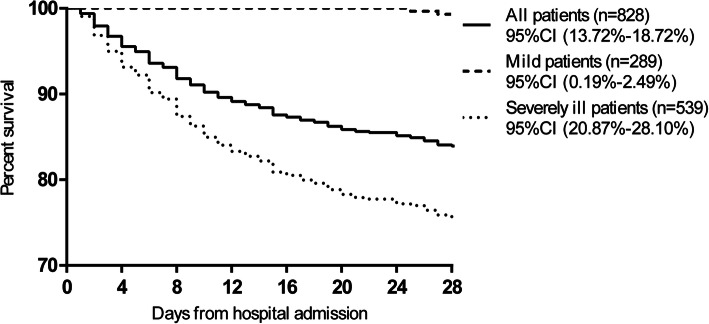
Kaplan-Meier survival curves for all the 828 patients and the two groups defined by the severity of illness

The treatments, outcomes, and complications of the 828 cases were shown in Table 2. A total of 681 (82.25%) patients received oxygen therapy, 149 (18.00%) patients received mechanical ventilation, and 75 (9.06%) patients received invasive mechanical ventilation. Antiviral therapies were used in 739 (89.25%) patients, systematic corticosteroids in 375 (45.29%) patients, and hydroxychloroquine in 57 (6.88%) patients. As of May 18, 2020, 682 (82.37%) patients have been discharged and 146 (17.63%) patients died. The median duration from illness onset to death in 146 deceased patients was estimated to be 20.0 days (IQR, 14.0–26.0). Figure 2 shows the 28-day Kaplan-Meier survival curves for all patients and the two subgroups categorized by the severity of illness. Of 146 non-survivors, 143 (97.95%) of the non-survivors developed ARDS; the most common complication was acute cardiac injury (40, 27.40%) followed by acute renal injury (32, 21.92%), septic shock (31, 21.23%), and acute liver injury (15, 10.27%).

**Table 2:** Treatment and complications of 828 patients with COVID-19

Characteristics		Enrolled patients (*n* = 828)	Training cohort (*n* = 516)	External validation cohort 1 (*n* = 186)	External validation cohort 2 (*n* = 126)
**Treatment**					
Antiviral therapy	*n* (%)	739 (89.25%)	448 (86.82%)	171 (91.94%)	120 (95.24%)
Arbidol	*n* (%)	595 (71.86%)	384 (74.42%)	161 (86.56%)	50 (39.68%)
Oseltamivir	*n* (%)	144 (17.39%)	88 (17.05%)	14 (7.53%)	42 (33.33%)
Remdesivir	*n* (%)	18 (2.17%)	12 (2.33%)	4 (2.15%)	2 (15.9%)
Ritonavir/lopinavir	*n* (%)	143 (17.27%)	98 (18.99%)	33 (17.74%)	12 (9.52%)
Antibiotics	*n* (%)	657 (79.35%)	416 (80.62%)	123 (66.13%)	118 (93.65%)
Antifungal therapy	*n* (%)	34 (41.06%)	24 (4.65%)	9 (4.84%)	1 (0.79%)
Corticosteroids	*n* (%)	375 (45.29%)	201 (38.95%)	71 (38.17%)	103 (81.75%)
Gamma globulin	*n* (%)	228 (27.54%)	121 (23.45%)	25 (13.44%)	82 (65.08%)
Hydroxychloroquine	*n* (%)	57 (6.88%)	37 (7.17%)	7 (3.76%)	13 (10.32%)
Vasopressors	*n* (%)	153 (18.48%)	91 (17.64%)	37 (19.89%)	25 (19.84%)
Oxygen therapy	*n* (%)	681 (82.25%)	421 (81.59%)	160 (86.02%)	100 (79.37%)
Mechanical ventilation	*n* (%)	149 (18.00%)	90 (17.44%)	32 (17.20%)	27 (21.43%)
Invasive mechanical ventilation	*n* (%)	75 (9.06%)	54 (10.47%)	19 (10.22%)	2 (1.58%)
Continuous renal replacement therapy	*n* (%)	32 (3.86%)	20 (3.88%)	11 (5.91%)	1 (0.79%)
**Outcomes**					
ICU admission	*n* (%)	100 (12.08%)	61 (11.82%)	27 (14.52%)	12 (9.52%)
Discharged	*n* (%)	682 (82.37%)	420 (81.40%)	156 (83.87%)	106 (84.13%)
Decreased	*n* (%)	146 (17.63%)	96 (18.60%)	30 (16.13%)	20 (15.87%)
Illness onset to death, days	Median (IQR)	20.0 (14.0–26.0)	20.0 (14.0–28.0)	22.0 (16.5–36.3)	21.0 (13.8–23.8)
Mortality rate	% (95% CI)	17.63% (15.19–20.38%)	18.60% (15.48–22.19%)	16.13% (11.54–22.09%)	15.87% (10.52–23.25%)
**Proximate causes of death in 146 patients**					
ARDS	143 (97.95%)				
Heart failure or acute cardiac injury	40 (27.40%)				
Acute renal injury	32 (21.92%)				
Acute liver injury	15 (10.27%)				
Septic shock	31 (21.23%)				
Acute pulmonary embolism	2 (1.37%)				
Gastrointestinal bleeding	5 (3.42%)

### Univariate and multivariable analyses for mortality in the training cohort

**Fig. 3:**
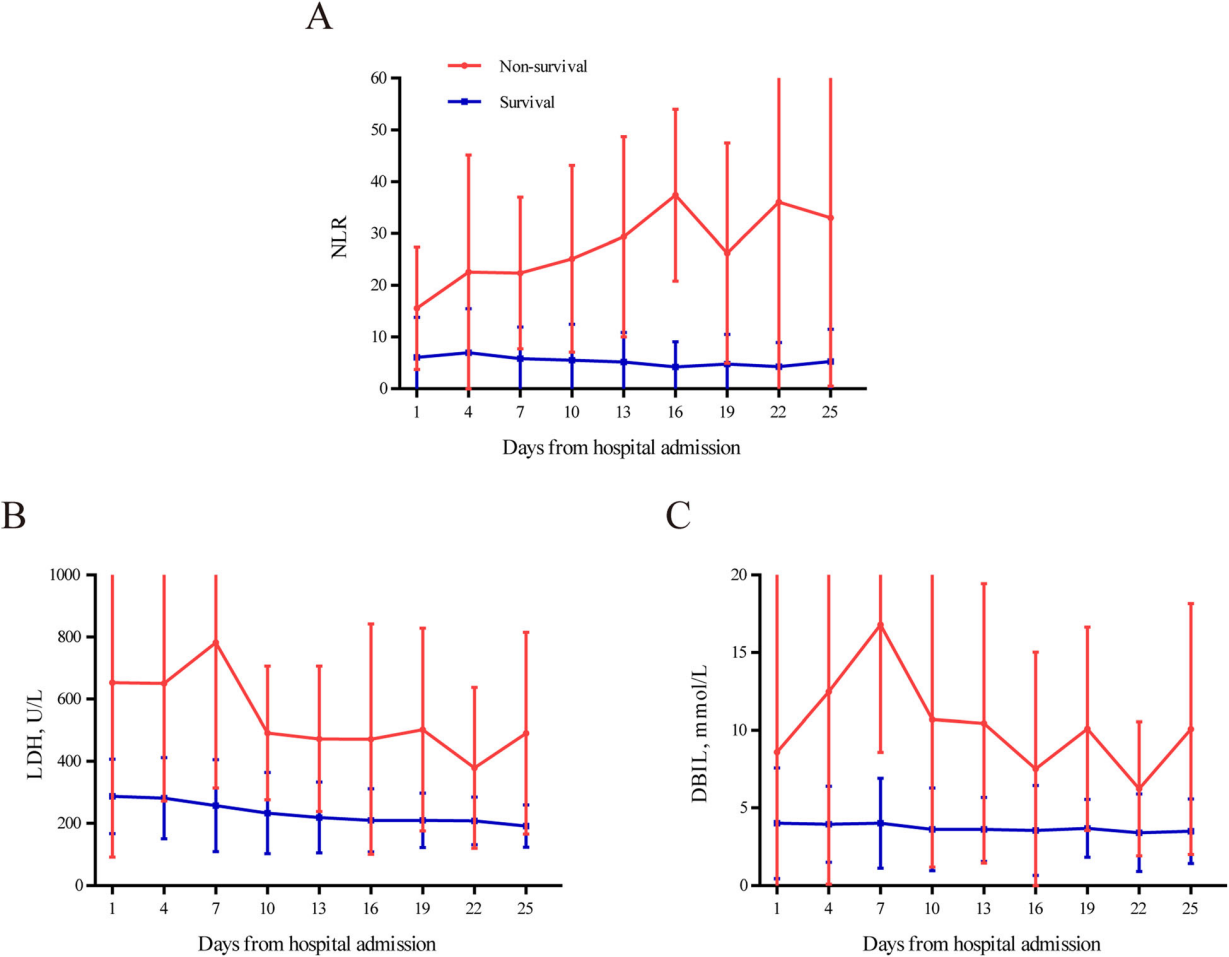
Temporal changes in the three independent laboratory risk factors from hospital admission in patients with COVID-19. Temporal changes in NLR (**a**). LDH (**b**). DBIL (**c**). Compared with survivors, non-survivors showed significant higher NLR, LDH, and direct bilirubin values in all time points

Next, we analyzed the risk factors for 28-day mortality in the training cohort by using Cox regression model. Eighty-seven decreased patients within 28 days were enrolled in the Cox regression analyses. Univariable Cox regression analyses showed age, male, dyspnea, respiratory rate, CURB-65 pneumonia severity score (CURB-65 score), quick Sepsis Related Organ Failure Assessment (qSOFA) score, reticular patterns, and 15 laboratory factors were associated with 28-day mortality (Table 3). The comparison between survivors and non-survivors in laboratory and CT findings were also displayed in Table S1 and Table S2. Multivariable Cox regression analyses showed that age (hazard ratio [HR], 1.05 [95% CI, 1.03–1.07]; *P* < 0.001), LDH level over 360 U/L (HR, 11.77 [95% CI, 5.62–24.65]; *P* < 0.001), NLR higher than 8.0 (HR 2.63, 95% CI [1.55–4.40]; *P* < 0.001), and DBIL higher than 5.0 μmol/L (HR 1.77, 95% CI [1.15–2.76]; *P* = 0.010) were associated with an increased likelihood of 28-day mortality (Table 4). Figure 3 showed the temporal changes of the three independent laboratory risk factors from hospital admission in survivors and non-survivors. Compared with survivors, non-survivors showed a significantly higher NLR, LDH, and DBIL value at all time points.

**Table 3:** Univariable Cox regression model for predicting 28-day mortality in 516 patients with COVID-19 at admission

Factors		Univariable HR (95% CI)	*P* value
Age, years#		1.05 (1.04–1.07)	< 0.001
Male sex (vs female)		2.76 (1.69–4.50)	< 0.001
Symptoms onset to admission, days#		0.98 (0.93–1.02)	0.293
Fever (yes vs no)		0.85 (0.46–1.55)	0.587
Dyspnea (yes vs no)		1.79 (1.17–2.74)	0.008
Comorbidity (yes vs no)		1.41 (0.93–2.15)	0.108
Respiratory rate, breaths per min	≥ 24	3.15 (2.07–4.81)	< 0.001
	< 24	1 (ref)	
Reticular patterns (yes vs no)		2.83 (1.55–5.16)	< 0.001
CURB-65 score#		3.49 (2.81–4.33)	< 0.001
qSOFA score#		3.56 (2.44–5.19)	< 0.001
Leucocytes count, × 10^9^/L#		1.17 (1.13–1.21)	< 0.001
Lymphocyte count, × 10^9^/L#		0.12 (0.07–0.23)	< 0.001
Neutrophils count, × 10^9^/L#		1.04 (1.02–1.05)	< 0.001
NLR	> 8.0	9.74 (5.96–15.94)	< 0.001
	≤ 8.0	1 (ref)	
Platelets count, × 10^9^/L	< 125	3.94 (2.51–6.18)	< 0.001
	≥ 125	1 (ref)	
Hemoglobin, g/L#		1.01 (1.00–1.03)	0.038
Albumin, g/L#		0.87 (0.83–0.92)	< 0.001
Total bilirubin, μmol/L	> 20.0	5.69 (3.47–9.33)	< 0.001
	13.0–20.0	1.93 (1.14–3.26)	0.014
	< 13.0	1 (ref)	
Direct bilirubin, μmol/L	> 5.0	4.95 (3.22–7.60)	< 0.001
	≤ 5.0	1 (ref)	
ALT, U/L	> 40.0	1.49 (0.99–2.25)	0.057
	≤ 40.0	1 (ref)	
Urea nitrogen, mmol/L	> 8.2	9.65 (5.49–16.93)	< 0.001
	5.0–8.2	3.83 (2.19–6.72)	< 0.001
	< 5.0	1 (ref)	
d-dimer, mg/L	> 1.0	7.33 (3.61–14.88)	< 0.001
	0.5–1.0	2.97 (1.25–7.05)	0.014
	< 0.5	1 (ref)	
PT, s	> 16.0	4.41 (2.55–7.62)	< 0.001
	≤ 16.0	1 (ref)	
CRP, mg/L	> 40.0	19.96 (4.90–81.40)	< 0.001
	8.0–40.0	5.04 (1.15–22.17)	0.032
	< 8.0	1 (ref)	
Procalcitonin, ng/mL#		1.20 (1.06–1.36)	0.005
LDH level, U/L	> 360	23.67 (11.86–47.24)	< 0.001
	≤ 360	1 (ref)	

# per 1 unit increase

*HR* hazard ratio, *ref* reference, *CURB-65* CURB-65 Score for Pneumonia Severity, *qSOFA* quick Sepsis-Related Organ Failure Assessment, *NLR* neutrophil-to-lymphocyte ratio, *LDH* lactate dehydrogenase, *ALT* alanine aminotransferase, *CRP* C-reactive protein, *PT* prothrombin time, *CI* confidence interval

**Table 4:** Independent risk factors of 28-day mortality and nomogram score

Factors		Multivariable HR (95% CI)	*P* value	Nomogram score
Age, years#		1.05 (1.03–1.07)	< 0.001	(Age-20) × 1.25
NLR	> 8.0	2.63 (1.55–4.40)	< 0.001	26.60
	≤ 8.0	1 (ref)		0
Direct bilirubin, μmol/L	> 5.0	1.77 (1.15–2.76)	0.010	16.06
	≤ 5.0	1 (ref)		0
LDH level, U/L	> 360	11.77 (5.62–24.65)	< 0.001	67.63
	≤ 360	1 (ref)		0

# per 1 unit increase

*HR* hazard ratio, *ref* reference, *NLR* neutrophil-to-lymphocyte ratio, *LDH* lactate dehydrogenase, *CI* confidence interval

### Development and validation of nomogram for 14-day and 28-day mortality

**Fig. 4:**
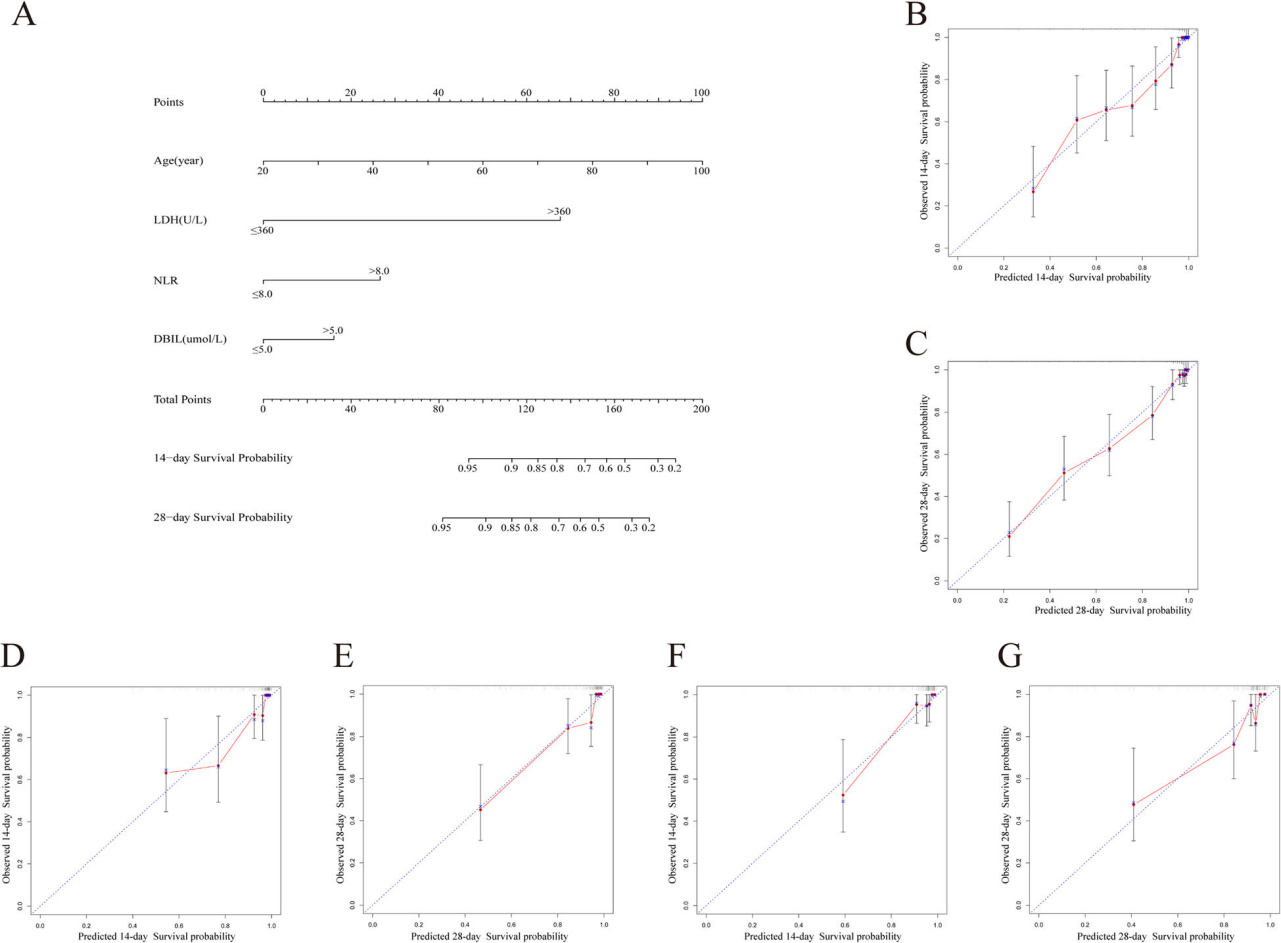
The nomogram scoring system for predicting patients’ survival probability based on age, LDH level, DBIL, and NLR. **a** Nomogram for predicting the probability of 14-day and 28-day survival. The number of points for each factor is in the top row. For each factor, the absence is assigned 0 points. The presence of factors is associated with the number of points. The points for each factor are summed together to generate a total point score. The total points correspond to the respective 14-day and 28-day survival probabilities. The ability of this model to distinguish between low-risk and high-risk patients can be demonstrated by considering two hypothetical individuals who might be encountered in practice: patient A is 60 years old with NLR of 10, DBIL of 4 μmol/L, and LDH of 400 U/L, getting a total score of 144.23; patient B is 40 years old with NLR of 3, DBIL of 10 μmol/L, and LDH 100 U/L, getting a total score of 41.06. Our model predicts that patient A’s 14-day survival probability is 75%, and his 28-day survival probability is 63%. For patient B, his 14-day survival probability and 28-day survival probability are more than 95%. **b**–**g** The calibration plot of survival probabilities at 14 days and 28 days. Nomogram-predicted survival probability is plotted on the *x*-axis, with observed survival probability on the *y*-axis. Dashed lines along the 45° line through the origin point represent the perfect calibration models in which the predicted probabilities are identical to the actual probabilities. The training cohort calibration plot of survival probabilities at 14 days (**b**) and 28 days (**c**). **d**, **e** The external validation cohort 1 calibration plot of survival probabilities at 14 days (**d**) and 28 days (**e**). **f**, **g** The external validation cohort 2 calibration plot of survival probabilities at 14 days (**f**) and 28 days (**g**)

Next, we worked out a nomogram scoring system for predicting the 14-day and 28-day survival probability of patients with COVID-19 on the basis of the four independent predictors of mortality (Fig. 4a). To help physicians better understand the scoring system, we explained how to calculate the score in the legend of Fig. 4. Figure 4b and c shows the calibration plot for the prediction model, in which the predicted probability of 14-day and 28-day survival is plotted against the observed data. The curves of predictive 14-day and 28-day survival probability were closely approximated to the observed probability, which means the nomogram scoring system exhibited good calibration. The discrimination of the constructed nomogram was evaluated with the C-index (0.886, 95% CI, 0.873–0.899), suggesting a favorable discriminative power. We also compared the nomogram score in our study with the CURB-65 score and qSOFA score. In the training cohort, the discrimination C-index of CURB-65 and qSOFA scores were 0.781 (95% CI, 0.757–0.805) and 0.672 (95% CI, 0.644–0.699), respectively. As indicated by the lack of overlap in the confidence intervals, the discrimination power of the nomogram score developed in the training cohort was significantly higher than that of the CURB-65 and qSOFA scores.

To further verify the nomogram scoring system, two external cohorts were included. The external validation cohort 1 was performed by using the Union Hospital external validation set. In the Union Hospital external validation set, the final multivariable model for 28-day mortality showed strong external validity, with a discrimination C-index of 0.879 (95% CI, 0.856–0.900) indicating an 87.9% correct model identification of the 28-day survival probability across all possible pairs of patients. In the Central Hospital of Wuhan validation set, the nomogram also exhibited a good discrimination power (C-index, 0.839, 95% CI [0.798–0.880]). Calibration of the nomogram predicted 14-day and 28-day survival probability corresponding with the actual survival in both external validation cohorts (Fig. 4d–g).

## Discussion

In this study, we employed the clinical and laboratory features of COVID-19 patients to work out an effective and easy tool for predicting 28-day mortality. Univariate analyses revealed that these factors including age, male sex, dyspnea, respiratory rate, CURB-65 score, qSOFA score, reticular patterns, leukocyte count, lymphocyte count, NLR, and several other biochemical parameters were associated with mortality. Multivariate analyses found that older age, NLR over 8.0, DBIL levels higher than 5.0 μmol/L, and LDH levels higher than 360 U/L at admission were four independent predictors of 28-day mortality in adult hospitalized patients with COVID-19.

Many more patients developed fever and had comorbidities including hypertension and diabetes than those in Guan et al.’s study with a relatively large sample size [7]. However, patients in our study were all from Wuhan city, while patients in Guan et al.’s study were from 30 provinces, autonomous regions, and municipalities in mainland China. Since a great shortage of medical resources existed in Wuhan city, the hardest-hit area of the COVID-19 outbreak at the early stage of this pandemic, this regional difference should be noted. When compared with other studies, patients in which were also from Wuhan, the proportions of patients with fever and comorbidities were comparable [4, 25]. The overall crude mortality rate in our series was higher than that in the previous report [26]. On the basis of a statistical model involving 72,314 patients, Zhong and his colleagues estimated that the case mortality rate was 2.3% in patients with confirmed COVID-19, 2.9% in Hubei province, and 49% in severely ill patients. However, Shang et al. reported that the mortality rate in severely ill patients with COVID-19 was about 49% [27]. The discrepancies in the mortality rates might be ascribed to proportions of patients of different severities in different cohorts, given that all death events in our cohort were observed in severely ill patients. Thus, the proportion of severe cases in our study should be taken into account. In fact, after a mandatory hierarchical management was introduced, more severe COVID-19 patients were transferred to our hospitals, while mild cases were re-directed to the “mobile cabin hospitals.”

Compared with survivors, more non-survivors were older, male, and were complicated with more chronic conditions. This result was coincident with the finding of a previous study focusing on critically ill COVID-19 patients [27]. As aforementioned, all the non-survivors except two were those who were categorized as severely ill at admission in our study. This result suggested that mild patients could be treated by home quarantine or in our mobile cabin hospitals, given their satisfactory survival and the shortage of medical resources. Of note, reticular patterns were more frequently found on CT images at presentation in non-survivors and were reportedly the predominant imaging finding on CT images 3 weeks after symptom onset [28].

Previous studies have reported that older male patients were more subject to COVID-19 infection, and severe patients were older than their non-severe counterparts [4, 7]. Compared with survivors, non-survivors were reported to be older in two observational studies [9, 27]. In this study, we found that age was an independent risk factor for 28-day mortality in patients with COVID-19. A higher level of LDH was suggested to indicate more extensive lung tissue injury and reported to be linked with poor outcomes in patients with severe acute respiratory syndrome (SARS) [25, 29]. In patients with COVID-19, plasma LDH level was reported to be higher in severe, ICU, and deceased COVID-19 patients than in mild, non-ICU-patients, and survivors [27, 30]. Our study showed that LDH could serve as a valuable predictor of mortality in COVID-19 patients, with its hazard ratio being the highest. Reminiscent of a previous mortality prediction model developed by Yan et al., LDH higher than 365 U/L was also reported to be a risk factor for mortality in patients with COVID-19 [31]. Meanwhile, this previous model highlighted the crucial role of LDH in distinguishing the vast majority of cases that required immediate medical attention. The elevated NLR was observed in patients who died of COVID-19 and found to be able to predict severe cases of COVID-19 at its early stage [20, 21]. This study confirmed that it could act as a predictor of mortality in COVID patients. DBIL was reported to be associated with severe COVID-19 in a multicenter retrospective study [19], now identified as one independent risk factor for 28-day mortality. Although the presence of preexisting comorbidities seems to increase the odds of death, the association was not significant in our study.

We also employed the four independent predictors to construct a predictive model which was shown in a form of nomogram scoring system. Our prediction mode was constructed based on a reasonable size and consecutive cohort of adult patients with confirmed COVID-19. This kind of sample selection minimized the selection bias. However, the proportion of severely ill patients was large in our hospital since Wuhan Union Hospital was a designated hospital for severely ill COVID-19 patient treatment. This made the cohort in our study less representative of adult hospitalized patients with confirmed COVID-19 in Wuhan. However, it should be highlighted that our model not only showed good discrimination and calibration in an external validation from the same hospital, but also performed well in an external validation cohort consisting of patients from another hospital, which was not a designated hospital for severely ill COVID-19 patient treatment. Therefore, our prediction models are based on and validated in Wuhan hospitalized populations with COVID-19 infection and should therefore be applicable to other sites within Wuhan. Compared with the CURB-65 and qSOFA scores, our scoring system displayed better discrimination ability in the training cohort. By employing our model, once the target patients’ data on the four risk factors were measured at admission, their risk of 14-day and 28-day mortality can be calculated by our model to guide the decision of clinical physicians. Considering that the outcome events outside Wuhan are different, when trying to apply this prediction mode into other provinces in China or other countries, this mode might need to be updated and adjusted to the local setting before it can safely be applied.

Our study had several limitations. First, it was of retrospective nature, and all data were collected from case records. Therefore, important information might be missed and further prospective studies are needed. Second, it is worth pointing out that the amount of missing data differed between the survivor and non-survivor groups, especially for ferritin and d-dimer. Even though we believe these differences were attributed to different physicians’ decisions in their clinical practice due to the absence of guideline recommendations, the resulting potential bias should be noted and further prospective studies can be also helpful to decrease this discrepancy in missing data. Third, this study included a high population of patients who were severely ill; there may be a selection bias when identifying the risk factors of mortality. Since physicians should evaluate the patients’ condition at admission, we focused on the information of patients at admission, other important factors during hospitalization that might influence case mortality, such as the use of non-invasive assisted ventilation or other medications and timing, as well as longitudinal observations of clinical and laboratory variables, were not covered. More detailed analyses involving these factors should be undertaken.

## Conclusions

In conclusion, our study demonstrated that older age, high lactate dehydrogenase level, evaluated neutrophil-to-lymphocyte ratio, and high direct bilirubin level were independent predictors of 28-day mortality in adult hospitalized patients with confirmed COVID-19. The new nomogram scoring system for the prediction of 14-day and 28-day survival probability based on the four variables showed good discrimination and calibration in two independent validation cohorts, suggesting a potential to guide the medical practitioners in the monitoring and management of COVID-19.

## Supplementary information

**Additional file 1.** Supplementary tables.

## Data Availability

The data that support the findings of this study are available from the corresponding authors upon reasonable request.

## Abbreviations

COVID-19: Coronavirus disease 2019
SOFA: Sequential Organ Failure Assessment
CRP: C-reactive protein
LDH: Lactate dehydrogenase
EPP: Candidate predictor parameter
DBIL: Direct bilirubin
NLR: Neutrophil-to-lymphocyte ratio
ALT: Alanine aminotransferase
PT: Prothrombin time
SD: Standard deviation
IQR: Interquartile range
CI: Confidence interval
HR: Hazard ratio
AST: Aspartate aminotransferase
GGT: Gamma-glutamyl transferase
APTT: Activated partial thromboplastin time
INR: International normalized ratio
