# Supplementary material for: Development and validation of a risk factor-based system to predict short-term survival in adult hospitalized patients with COVID-19: a multicenter, retrospective, cohort study
Source: Crit Care. 2020 Jul 16;24:438. doi: 10.1186/s13054-020-03123-x (PMC7364297; doi:10.1186/s13054-020-03123-x)
Supplement: Supplementary file 1 — Additional file 1. Supplementary tables. [file 13054_2020_3123_MOESM1_ESM.docx]

**Table S1 Laboratory findings of 516 patients with COVID-19 in training cohort at admission**

| **Characteristics** | **Survivors**  (n=420) | **Non-survivors**  (n=96) | **p value** |
| --- | --- | --- | --- |
| Leucocytes count, × 10^9^/L | 5.32 (4.14-7.18) | 8.13 (6.12-10.93) | <0.001* |
| Neutrophils count, × 10^9^/L | 3.77 (2.60-5.73) | 7.20 (5.13-9.74) | <0.001* |
| Lymphocyte count, × 10^9^/L | 0.97 (0.71-1.36) | 0.58 (0.42-0.82) | <0.001* |
| NLR | 3.91 (2.07-6.79) | 10.99 (7.68-20.97) | <0.001* |
| Hemoglobin, g/L | 127.00 (117.00-137.00) | 131.00 (119.00-144.00) | 0.060* |
| Platelets count, × 10^9^/L | 222.54 (88.27) | 169.77 (85.48) | <0.001† |
| Total bilirubin, μmol/L | 10.45 (7.83-13.90) | 15.05 (10.53-21.58) | <0.001* |
| Direct bilirubin, μmol/L | 3.35 (2.50-4.50) | 5.30 (4.03-8.68) | <0.001* |
| Albumin, g/L | 31.25 (27.90-34.45) | 27.90 (25.70-30.00) | <0.001* |
| Globin, g/L | 31.05 (5.77) | 33.62 (6.94) | 0.001† |
| ALT, U/L | 33.00 (20.00-54.00) | 40.00 (24.00-56.00) | 0.011* |
| AST, U/L | 32.00 (23.00-47.00) | 47.00 (35.00-65.00) | <0.001* |
| Urea nitrogen, mmol/L | 4.30 (3.26-5.94) | 6.47 (4.99-9.48) | <0.001* |
| Creatinine, μmol/L | 66.15 (56.60-79.55) | 75.10 (62.43-93.58) | <0.001* |
| GGT, U/L | 48.74 (64.33) | 64.67 (58.79) | 0.027† |
| LDH, U/L | 260.00 (205.00-335.00) | 556.00 (408.25-729.75) | <0.001* |
| CRP, mg/L | 25.92 (7.04-65.13) | 79.58 (43.33-124.18) | <0.001* |
| D-dimer, mg/L | 0.51 (0.27-1.46) n=344 | 3.59 (0.73-8.00) n=82 | <0.001* |
| PT, s | 13.30 (12.60-14.18) n=368 | 14.30 (13.23-15.38) n=92 | <0.001* |
| APTT, s | 37.05 (33.43-40.90) n=368 | 36.95 (32.95-43.68) n=92 | 0.446* |
| INR | 1.09 (0.64) n=368 | 1.16 (0.21) n=92 | 0.314† |
| Fibrinogen, g/L | 4.35 (3.51-5.00) n=368 | 4.40 (3.13-5.24) n=92 | 0.739* |
| Ferritin, ng/ml | 697.07 (536.01) n=132 | 1169.81 (609.51) n=25 | <0.001† |
| Creatine kinase, U/L | 71.00 (47.00 -137.25) n=328 | 139.50 (76.25-311.25) n=74 | <0.001* |
| ESR, mm/h | 47.00 (27.00-78.00) n=135 | 53.00 (25.50-77.50) n=25 | 0.851* |
| Procalcitonin, ng/ml | 0.09 (0.06-0.17) n=285 | 0.30 (0.14-0.50) n=54 | <0.001* |

Continuous variables were expressed as mean (SD) if they were normally distributed or median (IQR) if they were not.

Abbreviations: NLR, Neutrophil-to-lymphocyte ratio; ALT, Alanine aminotransferase; AST, Aspartate aminotransferase; LDH, Lactate dehydrogenase; GGT, Gamma-Glutamyl Transferase; CRP, C-reactive protein; PT, Prothrombin time; APTT, Activated partial thromboplastin time; INR, International normalized ratio.

*Calculated using the Wilcoxon rank-sum test.

†Calculated using the unpaired t test.

**Table S2 Radiographic findings of 395 patients with COVID-19 in training cohort**

| **Characteristics** | **All patients** (n=395) | **Survivors** (n=337) | **Non-survivors** (n=58) | **p value** |
| --- | --- | --- | --- | --- |
| Patchy and ground-glass opacity | 375 (94.49%) | 321 (95.25%) | 54 (93.10%) | 0.491 ‡ |
| Involved lungs |  |  |  |  |
| Unilateral | 32 (8.10%) | 28 (8.31%) | 4 (6.90%) | 0.716§ |
| Bilateral | 363 (91.90%) | 309 (91.69%) | 54 (93.10%) | - |
| Reticular patterns | 42 (10.63%) | 28 (8.31%) | 14 (24.14%) | <0.001‡ |
| Consolidation | 128 (30.33%) | 113 (33.53%) | 15 (25.86%) | 0.249‡ |
| Thickening of the adjacent pleura | 45 (11.00%) | 40 (11.87%) | 5 (8.62%) | 0.654§ |
| Interlobular septal thickening | 13 (3.29%) | 10 (2.97%) | 3 (5.17%) | 0.417§ |
| Pleural effusion | 25 (6.33%) | 24 (7.12%) | 1 (1.72%) | 0.150§ |
| Lymphadenopathy | 30 (7.59%) | 28 (8.31%) | 2 (3.45%) | 0.284§ |

Categorical variables were presented as frequency rates and percentages.

Continuous variables were expressed as mean (SD) if they were normally distributed or median (IQR) if they were not.

‡Calculated using the χ2 test.

§Calculated using the Fisher’s exact test.
